# PMAnalyzer: a new web interface for bacterial growth curve analysis

**DOI:** 10.1093/bioinformatics/btx084

**Published:** 2017-02-13

**Authors:** Daniel A Cuevas, Robert A Edwards

**Affiliations:** 1 Computational Science Research Center, San Diego State University, San Diego, CA, USA; 2 Department of Computer Science, San Diego State University, San Diego, CA, USA

## Abstract

**Summary:**

Bacterial growth curves are essential representations for characterizing bacteria metabolism within a variety of media compositions. Using high-throughput, spectrophotometers capable of processing tens of 96-well plates, quantitative phenotypic information can be easily integrated into the current data structures that describe a bacterial organism. The *PMAnalyzer* pipeline performs a growth curve analysis to parameterize the unique features occurring within microtiter wells containing specific growth media sources. We have expanded the pipeline capabilities and provide a user-friendly, online implementation of this automated pipeline. *PMAnalyzer* version 2.0 provides fast automatic growth curve parameter analysis, growth identification and high resolution figures of sample-replicate growth curves and several statistical analyses.

**Availability and Implementation:**

*PMAnalyzer* v2.0 can be found at https://edwards.sdsu.edu/pmanalyzer/. Source code for the pipeline can be found on GitHub at https://github.com/dacuevas/PMAnalyzer. Source code for the online implementation can be found on GitHub at https://github.com/dacuevas/PMAnalyzerWeb.

**Supplementary information:**

Supplementary data are available at *Bioinformatics* online.

## Introduction

Bacteria growth curves have been proven useful in several studies where cellular proliferation is the primary measured response; for example, in characterizing novel bacteriophage proteins (Sanchez *et al.*, 2015), in describing mutant bacterial strains (Perkins and Nicholson, 2008) and in elucidating genome-scale metabolic models (Cuevas *et al.*, 2016a,b; Kim and Reed, 2014). Automated multi-96-well plate spectrophotometer systems provide the ability to test hundreds of these hypotheses at a time, monitoring biomass accumulation over a span of hours or days.

Downstream analytical tools need to (i) easily and quickly process datasets in various formats, (ii) provide transparency and clear explanations of their pipeline and (iii) produce results flexible enough to become integrated with other quantitative information. Software capable of decomposing growth experimental data into usable metrics, such as the carrying capacity of an organism expressed in various growth media, is not freely and easily accessible. Specifically with 96-well plates, this type of software is normally developed for input of a single data format (e.g. GCAT (Bukhman *et al.*, 2015)), to serve a specific spectrophotometer technology, or requires some programming knowledge to run and diagnose (Vehkala *et al.*, 2015).

We present an improved version of our growth curve processing pipeline, *PMAnalyzer* v2.0, extended to include a variety of growth metrics, statistical results and a user-friendly online interface. The *PMAnalyzer* web tool allows users to analyze their growth curve data, view their results and export their information for subsequent analyses.

## 2 Materials and methods

### 2.1 Bacterial growth curve model

Each bacterial growth curve is numerically fitted to the Zwietering logistic growth curve model (Zwietering *et al.*, 1990) using a least-squares fitting on the raw data:

$$\hat{y}=y_{\text{0}}+\frac{A-y_{0}}{1+\exp\left[\left(\frac{4\mu }{A}\right)\left(\lambda -t\right)+2\right]}$$

The model provides four key parameters that describe a growth curve: starting absorbance value $y_{0}$ (OD 600 nm), lag time λ (hour), maximum growth rate μ (OD 600 nm per hour) and biomass yield *A* (OD 600 nm). The parameter *t* is the time vector (hour). The result, $\hat{y}$, is a vector of logistic-fitted values. Each growth curve is modeled with this formula independent of technical and biological replicates. Afterwards, the four parameters are averaged between a sample’s replicates to form a representative logistic-fitted growth curve of that sample. Raw growth curves, individual fitted growth curves, and representative fitted growth curves are all supplied in separate plain text files by the *PMAnalyzer*.

### 2.2 Growth analysis

The growth level metric, recapitulated from the previous version of *PMAnalyzer*, represents a single quantitative value of growth (Cuevas *et al.*, 2016a). New metrics included in this updated version are: scaled growth level, growth class and mean squared error of each logistic fitting. Area under the curve calculations are also provided for raw data, normalized data, logistic fitted raw data and logistic fitted normalized data. For the complete list of the output metrics and their explanations, see the results documentation in the Supplementary Material.

## 3 Web implementation

### 3.1 Usage

The online implementation requires the user to input only a minimal amount of information. This updated version of *PMAnalyzer* is capable of processing datasets that have been generated in three unique formats, each defined from various projects using the pipeline. Each of those formats has been extensively described in *PMAnalyzer*’s help webpage (https://edwards.sdsu.edu/pmanalyzer/help.html).

Most multi-plate spectrophotometers generate a separate data file of absorbance data for each plate. The user must uniquely name each file with a representative name and replicate identifier. This allows *PMAnalyzer* to perform various statistical analyses in regards to each sample and replicate.

Plate media information is optionally supplied to *PMAnalyzer*. The user has the option to either choose a pre-configured plate file (descriptions supplied in the help webpage), supply their own plate configuration file, or not use one at all. Without a plate configuration, *PMAnalyzer* differentiates environments based on the 96-well identifiers (e.g. A1, A2, B1, B2).

### 3.2 Job processing

During data processing, users are supplied a Job ID and a link to the log file. The Job ID is used to retrieve results in the future—this feature is supplied in the web tool, storing results for up to two weeks. The log file contains the verbose option output from the command-line. Here, users can view the current status of the job, what stage the job is at, and specific error messages, if any occur.

### 3.3 Results

At completion various tab-delimited plain text files are presented to be viewed or downloaded. Choosing to view a file opens a separate webpage where the contents of a file are presented in an HTML table for easy viewing.

**Fig. 1:**
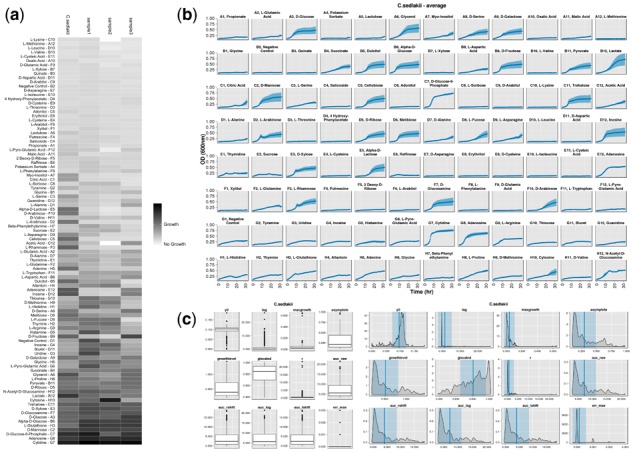
*PMAnalyzer* figure outputs. Figures generated after processing four different samples. (**a**) Comparative growth level heatmap between four samples. (**b**) Average growth curves of a *Citrobacter sedlakii* sample with standard error intervals. (**c**) Box and density plots of several metrics reported by the pipeline. Intervals indicate ±1 standard deviation

Several results images are generated showing from both the raw and processed data (Fig. 1). If more than one sample is processed in a single job, comparative figures are generated displaying each sample’s growth curves (averaged over a sample’s replicates), heatmaps of growth levels (Fig. 1a) and density plots of each metric as a whole. In addition to these comparative figures, similar figures are generated for each individual sample (Fig. 1b, c).

## Funding

This work is supported by the National Science Foundation [CNS-1305112, MCB-1330800].

*Conflict of Interest*: none declared.

## Supplementary Material

Supplementary Data
