# Supplementary material for: PMAnalyzer: a new web interface for bacterial growth curve analysis
Source: Bioinformatics. 2017 Feb 13;33(12):1905–6. doi: 10.1093/bioinformatics/btx084 (PMC5870709; doi:10.1093/bioinformatics/btx084)
Supplement: Supplementary Data [file btx084_supp.zip › PMAnalyzerOutput.pdf]

PMAnalyzer Output Files

PMAnalyzer version 2.0 --- document last updated October 24, 2016
Source code @ ***https://github.com/dacuevas/PMAnalyzer***

In each file, **[name]** is a custom suffix appended to the name of each file.

raw_curves_[name].txt
The raw data formatted for input into the pipeline. Columns include:

1. *sample* – sample identifier
2. *rep* – replicate identifier
3. *plate* – plate filename. “plate” is used if a file was not specified
4. *mainsource* – type of compound
5. *compound* – compound name
6. *well* – well identifier
7. *time* – time in hours
8. *od* – optical density reading

These column names are consistent among all growth curve files listed in this document.

---

logistic_curves_sample_[name].txt
The logistic curves fitted for each replicate. Logistic curves are obtained by using a least-squares curve fitting to the Zwietering logistic growth equation. Parameters are located in the “logistic params” file.

$$\hat{y} = y_0 + \frac{A - y_0}{1 + \exp\left[\frac{4\mu_{max}}{A}(\lambda - t) + 2\right]}$$

---

all_curves_mean_[name].txt
The mean growth curve for each well between replicates.

---

all_curves_median_[name].txt
The median growth curve for each well between replicates.

---

logistic_curves_mean_[name].txt
Logistic curve resulting from the logistic equation using the parameters from the “params_mean” file.

---

logistic_params_sample_[name].txt

The growth parameters used to create the logistic curves found in the “logistic curves” file. Columns include:

1. *sample* — sample identifier
2. *rep* — replicate identifier
3. *well* — well identifier
4. *mainsource* — type of compound
5. *compound* — compound name
6. *y0 [OD]* — starting optical density value (estimated by curve fitting)
7. *lag [t]* — lag time (estimated by curve fitting)
8. *maxgrowth [OD/time]* — maximum growth rate (estimated by curve fitting)
9. *asymptote [OD]* — carrying capacity or biomass yield (estimated by curve fitting)
10. *growthlevel [OD]* — a growth level calculation based on the harmonic mean equation using the logistic fitted values and asymptote

$$g = \frac{n}{\sum_i^n \frac{1}{(\hat{y}_i - y_0) + (A - y_0)}}$$

11. *glscaled [dimensionless]* — a growth level calculation based on the logistic fitted values, scaled by the asymptote. This value is somewhat similar to *growthlevel* but is scaled by the asymptote value, making it dimensionless. The minus one in the equation creates a metric between zero and one.

$$g_{scaled} = \frac{n}{A \sum_i^n \frac{1}{\hat{y}_i + A}} - 1$$

12. *r [1/time]* — exponential growth rate

$$r = \frac{4\mu_{max}}{A}$$

13. *auc_raw [OD time]* — the area under the curve of the raw data up to the final time sampled. This is calculated using the composite trapezoidal rule (*scipy.integrate.trapz* SciPy function link).
14. *auc_rshift [OD time]* — the area under the curve (using calculation from *auc_raw*) while subtracting the area below the starting OD value

$$auc_{rshift} = auc_{raw} - y_0 t_f$$

15. *auc_log [OD time]* — the area under the curve of the logistic data up to the final time sampled. This is calculated using the integral.

$$auc_{log} = \int_0^{t_f} \frac{A - y_0}{1 + \exp\left[\frac{4\mu_{max}}{A}(\lambda - t) + 2\right]} dt$$

$$= \frac{A\,(A - y_0)\log\left(\exp\left[\frac{4\mu_{max}(\lambda - t_f)}{A} + 2\right] + 1\right)}{4\mu_{max}} + At_f$$

$$- \frac{A\,(A - y_0)\log\left(\exp\left[\frac{4\mu_{max}}{A} + 2\right] + 1\right)}{4\mu_{max}}$$

16. *auc_lshift [OD time]* — the area under the curve (using calculation from *auc_log*) while subtracting the area below the starting OD value (similar to 14).
17. *auc_log [OD time]* — the area under the curve of the logistic data up to the final time sampled. This is calculated using the integral.
18. *growthclass* — qualitative classification of growth based on *growthlevel*

$$class = \left\{ \begin{array}{cc} +++ & g \geq 0.75 \\ ++ & 0.50 \leq g < 0.75 \\ + & 0.35 \leq g < 0.50 \\ - & 0.25 \leq g < 0.35 \\ -- & g < 0.25 \end{array} \right\}$$

19. *err_mse* — the mean squared error of the curve fit to the real data, an indicator to how well the logistic fits the raw data

logistic_params_mean_[name].txt

The mean *y0*, *lag time*, *maximum growth rate*, and *asymptote* are calculated for each well within a sample (between replicates). For example, if a sample contains three replicates, then the mean maximum growth rate is calculated between the replicates

$$(\mu_{max}1 + \mu_{max}2 + \mu_{max}3) / 3$$

This is performed for each fitted growth parameter (i.e., *y0, lag, maxgrowth, asymptote*). The other metrics are not averaged (*growthlevel, r, auc,* etc.). They are recalculated using the logistic curve in the “logistic_curves_mean” file and/or mean growth parameters.

sample_statistics_[name].txt

Computed average, median, and standard deviation of each metric listed in the “params_sample” file. Each statistic is provided for each sample replicate, thus the statistic is computed over each well for a replicate.

well_statistics_[name].txt

Computed average, median, and standard deviation of each metric listed in the “params_sample” file. Each statistic is provided for each well, thus the statistic is computed over all samples and replicates.
